# Supplementary material for: School-Based Physical Activity Interventions in Children and Adolescents: A Systematic Review
Source: Int J Environ Res Public Health. 2020 Feb 5;17(3):999. doi: 10.3390/ijerph17030999 (PMC7037705; doi:10.3390/ijerph17030999)
Supplement: Supplementary file 1 [file ijerph-17-00999-s001.pdf]

Supplementary Material

School-based Physical Activity Interventions in Children and Adolescents: A Systematic Review

Mikel Vaquero-Solís [1], Damián Iglesias Gallego [1,*], Miguel Ángel Tapia-Serrano [1], Juan J. Pulido [2,3] and Pedro Antonio Sánchez-Miguel [1,*]

[1] Department of Didactics of Music, Plastic and Body Expression, Teacher Training College, University of Extremadura, Cáceres (Spain), Extremadura, 10003, Spain; mivaquero@alumnos.unex.es (M.V.-S); matapiase@unex.es (M.A.T.-S.)
[2] University of Extremadura, Faculty of Sport Science, Cáceres (Spain), Extremadura, 10003, Spain; jjpulido@unex.es (J.J.P.)
[3] Faculty of Human Kinetics, University of Lisbon, Lisbon 1499-002, Portugal
* Correspondence: pesanchezm@unex.es (P.A.S.-M.); diglesia@unex.es (D.I.G) Tel.: (+34-927-257-647)

Supplementary Table S1

Theoretical Concepts	Intervention Variables	Study (Year)[Reference]	Sizes Effects × time (η^2; η^2p; Clif delta; Cohen's *d*; R^2)	Sizes Effects Between Groups (η^2, Clif delta, Cohen's *d*; R^2)
Motivation	Intrinsic Regulation	Cuevas et al. (2016)[48]	$\eta^2 = 0.075$	
		Fernández-Rio et al. (2016)[49]	Cohen's $d = 0.12$	
		Franco and Coteron (2017)[50]	Cliff's delta = 0.10	
		Lubans et al. (2016)[64]	Cohen's $d = 0.53$	
		Nicaise et al. (2013)[65]	Cohen's $d = 0.02$	
		González-Cutre et al. (2016)[53]	Cliff's delta = 0.51	
		Sevil et al. (2015)[74]		$\eta^2p = 0.62$
		Sevil et al. (2016)[75]		$\eta^2p = 0.061$
		Sevil et al. (2018)[76]		$\eta^2p = 0.165$
	Integrated Regulation	González-Cutre et al. (2016) [54]	Cliff's delta = 0.36	
	Identified Regulation	Amado et al. (2014)[21]	$\eta^2 = 0.08$	
		Cuevas et al. (2016)[48]	$\eta^2 = 0.056$	
		Fernández-Rio et al. (2016)[49]	Cohen's $d = 0.17$	
		González-Cutre et al. (2014)[53]	Cliff's delta = 0.54	

Supplementary Table S1. *Cont.*

		González-Cutre et al. (2016)[55]	Cliff's delta = 0.53	
		Lubans et al. (2016)[64]	Cohen's d = 0.40	
		Nicaise et al. (2014)[65]	Cohen's d = 0.26	
		Sevil et al., (2016)[75]		$\eta^2 p$ = 0.034
		Sevil et al. (2018)[76]		$\eta^2 p$ = 0.092
	Autonomous Motivation	Bechter et al. (2019)[44]	Cohen's *d* = 0.11	
		Cuevas et al. (2016)[48]	η^2 = 0.050	
		Perlman et al. (2013)[67]	η^2 *p* = 0.157	
		Piipari et al. (2018)[68]	η^2 = 0.06	
		Riiser et al. (2014)[40]	Cohen's *d* = 0.11	
		Sánchez-Oliva et al. (2017)[73]	η^2 = 0.08	
		Sevil et al. (2015)[74]		$\eta^2 p$ = 0.62
	Interjected Regulation	González-Cutre et al. (2014)[53]	Cliff's delta = 0.38	
		Lubans et al. (2016)[64]	Cohen's *d* = 0.56	
	External Regulation	Fernández-Rio et al. (2016)[49]	Cohen's *d* = 0.03	
		González -Cutre et al. (2014)[53]	Cliff's delta = 0.20	
		Lubans et al. (2016)[64]	Cohen's *d* = 0.49	
		Nicaise et al. (2014)[65]	Cohen's *d* = 0.32	
	Controlled Motivation	Sánchez-Oliva et al. (2017)[73]	(η^2 = 0.06)	
	Amotivation	Cheon et al. (2016)[22]	N/A	
		Kanlanyanee et al. (2017)[59]	N/A	
Need satisfaction	Competence	Bechter et al. (2019)[44]	Cohen's *d* = 0.08	
		Franco and Coteron (2017)[50]	Cliff's delta = 0.38	
		Sevil et al. (2015)[74]	$\eta^2 p$ = 0.034	
		Sevil et al. (2016)[75]		$\eta^2 p$ = 0.055
		Sevil et al. (2018)[76]	$\eta^2 p$ = 0.149	
		Tilga et al. (2019) [80]	$\eta^2 p$ = 0.03	
	Autonomy	Amado et al. (2014)[21]	η^2 = 0.08	
		Bechter et al. (2019)[44]	Cohen's *d* = 0.30	
		Franco and Coteron (2017)[80]	Cliff's delta = 0.31	
		González-Cutre et al. (2016)[54]	Cliff's delta = 0.45	

Supplementary Table S1. *Cont.*

		Sánchez-Oliva et al. (2017)[73]	($\eta^2 = 0.08$)	
		Sevil et al. (2016)[75]		$\eta^2 p = 0.022$
		Sevil et al. (2018)[76]		$\eta^2 p = 0160$
		Shanon et al. (2018)[77]	N/A	
		Tilga et al. (2019)[80]	$\eta^2\ p = 0.04$	
	Relatedness	Bechter et al. (2019)[44]	Cohen's $d = 0.22$	
		González-Cutre et al. (2016)[54]	Cliff's delta = 0.53	
		Tilga et al. (2019)[80]	$\eta^2 p = 0.03$	
		Cheon et al. (2018)[47]	Cohen's d = 0.56	
Need support	Competence	Abos et al. (2016)[41]	$\eta^2 p = 0.177$	
		Fu et al. (2016)[51]	N/A	
		Perlman et al. (2013)[67]	$\eta^2 p = 0.131$	
		Sevil et al. (2015)[74]	$\eta^2 p = 0.055$	
		Sevil et al. (2016)[75]		$\eta^2 p = 0.62$
		Sevil et al. (2018)[76]		$\eta^2 p = 0.088$
	Autonomy	Cheon et al. (2018)[47]	Cohen's $d = 0.99$	
		González-Cutre et al. (2014)[53]	Cliff's delta = 0.44	
		Piipari et al. (2018)[68]	$\eta^2 = 0.09$	
		Sánchez-Oliva et al. (2017)[73]	($\eta^2 = 0.08$)	
		Sevil et al. (2015)[74]		$\eta^2 p = 0.62$
		Sevil et al. (2018)[76]		$\eta^2 p = 0.088$
		Shanon et al. (2018)[77]	N/A	
	Relatedness	Sánchez-Oliva et al. (2017)[73]	($\eta^2 = 0.05$)	
		Sevil et al. (2015)[74]		$\eta^2 p = 0.62$
		Bronikowski et al. (2013)[46]	$\eta^2 = 0.58$	
Need frustration	Competence	Cuevas et al. (2016)[48]	$\eta^2 = 0.031$	
		Sevil et al. (2018)[76]		$\eta^2 p = 0.209$
	Autonomy	Sevil et al. (2018)[76]		$\eta^2 p = 0.192$
		Tilga et al. (2019)[80]	$\eta^2\ p = 0.04$	

Supplementary Table S1. *Cont.*

	Relatedness	Cuevas et al. (2016)[48]	$\eta^2 = 0.037$	
		Sevil et al. (2018)[76]		$\eta^2 p = 0.062$
	Frustration	Cheon et al., (2018)[47]	Cohen's $d = 0.63$	
Achievement Goal Theory	Task-oriented Climate	Bortoli et al. (2015)[45]	Cohen's $d = 0.80$	
		Grasten et al. (2015)[55]	$R^2 = 0.37$	
		Kokonen et al. (2018)[60]	$\eta^2 = 0.05$	
		Rokka et al. (2019)[72]	$\eta^2 = 0.04$	
		Sevil et al. (2016)[75]		$\eta^2 p = 0.090$
		Sevil et al. (2018)[76]		$\eta^2 p = 0.178$
	Ego-oriented Climate	Bortoli et al. (2015)[45]	Cohen's $d = 0.55$	
		Grasten et al. (2015)[55]	$R^2 = 0.16$	
		Kokonen et al. (2018)[60]	$\eta^2 = 0.02$	
		Rokka et al. (2019)[72]	$\eta^2 = 0.06$	
		Sevil et al. (2016)[75]		$\eta^2 p = 0.074$
		Sevil et al. (2018)[76]		$\eta^2 p = 0.036$
Behaviours	Intention to be Physically Active	Abos et al. (2016)[41]	$\eta^2 p = 0.365$	
		Cuevas et al. (2016)[48]	$\eta^2 =0.015$	
		Franco and Coteron (2017)[50]	Cliff's delta = 0.22	
		González-Cutre et al. (2014)[53]	Cliff's delta = 0.45	
		Rhodes et al. (2018)[70]	$R^2 = 0.41$	
		Sánchez-Oliva et al. (2019)[73]	($\eta^2 = 0.04$)	
		Sevil et al. (2018)[76])		$\eta^2 p = 0.064$
	Sedentary	Girelli et al. (2016)[52]	$\eta^2 p = 0.05$	
		Lonsdale et al. (2013)[62]	Cohen's $d = 0.72$	
		Lonsdale et al. (2017)[63]	Cohen's $d = 0.13$	
		Lubans et al. (2016)[64]	Cohen's $d = -32.2$	
	Lifestyle	Girelli et al. (2016)[52]	$\eta^2 p = 0.06$	
	Physical Activity	Bronikowski et al. (2013)[46]	$\eta^2 = 0.05$	
		Fu et al. (2016)[51]	N/A	
		González-Cutre et al. (2014)[53]	Cliff's delta = 0.53	

Supplementary Table S1. *Cont.*

		González-Cutre et al. (2016)[54]	Cliff's delta = 0.27	
		Grasten et al. (2018)[56]	R^2 = 0.34	
		Grasten et al. (2015)[55]	η^2 = 0.020	
		How et al. (2013)[58]	$\eta^2 p$ = 0.20	
		Lonsdale et al. (2013)[62]	Cohen's *d* = 0.45	
		Lonsdale et al. (2017)[63]	Cohen's *d* = 0.85	
		Nation Grainger et al. (2017)[79]	Small size < 0.01	
		Nicaise et al. (2014)[65]	Cohen's *d* = 0.07	
		Piipari et al. (2018)[68]	η^2 = 0.06	
		Riiser et al. (2014)[40]	Cohen's *d* = 0.55	
		Shanon et al. (2018)[77]	N/A	
	Attitude	Abos et al. (2016)[41]	$\eta^2 p$ = 0.251	
		Fernández-Rio et al. (2016)[49]	Cohen's d = 0.05	
		Girelli et al. (2016)[52]	$\eta 2p$ = 0.04	
		Gonzalez-Cutre et al. (2014)[53]	Cliff's delta = 0.34	
		González-Cutre et al. (2016)[54]	Cliff's delta = 0.34	
		Hajar et al. (2019)[57]	η^2 = 0.043	
		Sevil et al. (2015)[74]		$\eta^2 p$ = 0.62
		Tilga et al. (2019)[80]	$\eta 2\ p$ = 0.06	
	Effort	Bechter et al. (2019)[44]	Cohen's *d* = 0.30	
		Rokka et al. (2019)[72]	η^2 = 0.09	
	Behavioral Perceived Control	García-Calvo et al. (2015)[23]	N/A	
		Laroche et al. (2015)[61]	N/A	
	Engagement	Cheon et al. (2016)[22]		
Anthropometric variables	Cardiorespiratory Fitness	Lubans et al. (2016)[64]	Cohen's *d* = 5.9	
	BMI/Adiposity	Lubans et al. (2016)[64]	N/A	
		Riiser et al. (2014)[40]	Cohen's *d* = -0.70	
		Sebire et al. (2016)[39]	N/A	
		Smith et al. (2016)[78]	N/A	

Supplementary Table S1. *Cont.*

Psychosocial variables	Body Image	Riiser et al. (2014)[40]	Cohen's *d* = 0.56	
	Quality Life	Riiser et al. (2014)[40]	Cohen's *d* = 0.45	
		Shanon et al. (2018)[77]	$R^2 = 0.21$	
	Well-being	Babic et al. (2016)[43]	Small size < 0.01	
	Funny/Enjoy	Abos et al. (2016)[41]	$\eta^2\ p = 0.155$	
		Franco and Coteron (2017)[50]	Cliff's delta = 0.40	
		Fu et al. (2016)[51]	N/A	
		Hajar et al. (2019)[57]	$\eta^2 = 0.043$	
		Nicaise et al. (2014)[65]	Cohen's *d* = 0.99	
		Perlman et al. (2013)[67]	$\eta^2\ p = 0.122$	
		Sevil et al. (2015)[74]		$\eta^2 p = 0.62$
		Sevil et al. (2016)[75]		$\eta^2 p = 0.070$
		Sevil et al. (2018)[76]		$\eta^2 p = 0.180$
	Boredom	Cuevas et al. (2016)[48]	$\eta^2 = 0.050$	
		Sevil et al. (2015)[74]		$\eta^2 p = 0.31$
	Usefulness	Amado et al. (2017)[42]	N/A	
	Self-efficacy	Abos et al. (2016)[41]	$\eta^2 p = 0.111$	
		Bechter et al. (2019)[44]	Cohen's *d* = 0.41	
		Bronikowski et al. (2013)[46]	$\eta^2 = 0.10$	
		Tilga et al. (2019)[80]	$\eta^2 p = 0.04$	
	Appearance	Hajar et al. (2019)[57]	$\eta^2 = 0.043$	
	Psychological Condition	Hajar et al. (2019)[57]	$\eta^2 = 0.043$	
		Tilga et al. (2019)[80]	$\eta^2 p = 0.09$	
	Subjective Norms	González-Cutre et al. (2014)[53]	Cliff's delta = 0.34	
		Laroche et al. (2019)[61]	$R^2 = 0.09$	
	Social Support	Robbins et al., (2019)[71]	R = 0.28	
	Prosocial Behavior	Abos et al. (2016)[41]	$\eta^2 p = 0.144$	
		Bortoli et al. (2015)[45]	Cohen's *d* = 0.45	
		Cheon et al. (2018)[47]	Cohen's *d* = 0.56	
	Antisocial Behavior	Bortoli et al. (2015)[45]	Cohen's *d* = 0.64	
		Cheon et al. (2018)[47]	Cohen's *d* = 0.72	

	Sevil et al. (2018))[76]	$\eta^2 p = 0.149$
